# [*N*′-(5-Bromo-2-oxidobenzyl­idene-κ*O*)-3-hydr­oxy-2-naphthohydrazidato-κ^2^ *N*′,*O*]dibutyl­tin(IV)

**DOI:** 10.1107/S1600536809024477

**Published:** 2009-07-04

**Authors:** See Mun Lee, Kong Mun Lo, Hapipah Mohd Ali, Seik Weng Ng

**Affiliations:** a Department of Chemistry, University of Malaya, 50603 Kuala Lumpur, Malaysia

## Abstract

The Sn^IV^ atom in the title compound, [Sn(C_4_H_9_)_2_(C_18_H_11_BrN_2_O_3_)], shows a distorted *cis-*C_2_NO_2_Sn trigonal-bipyramidal coordination. One of the butyl chains is disordered over two sites in a 0.60 (1):0.40 (1) ratio.

## Related literature

The dianions of similar *N*′-(2-hydroxy­benzyl­idene)benzohydrazones *O*,*N*,*O*′-chelate to tin in organotin compounds; see: Labib *et al.* (1996 ▶); Samanta *et al.* (2007 ▶).
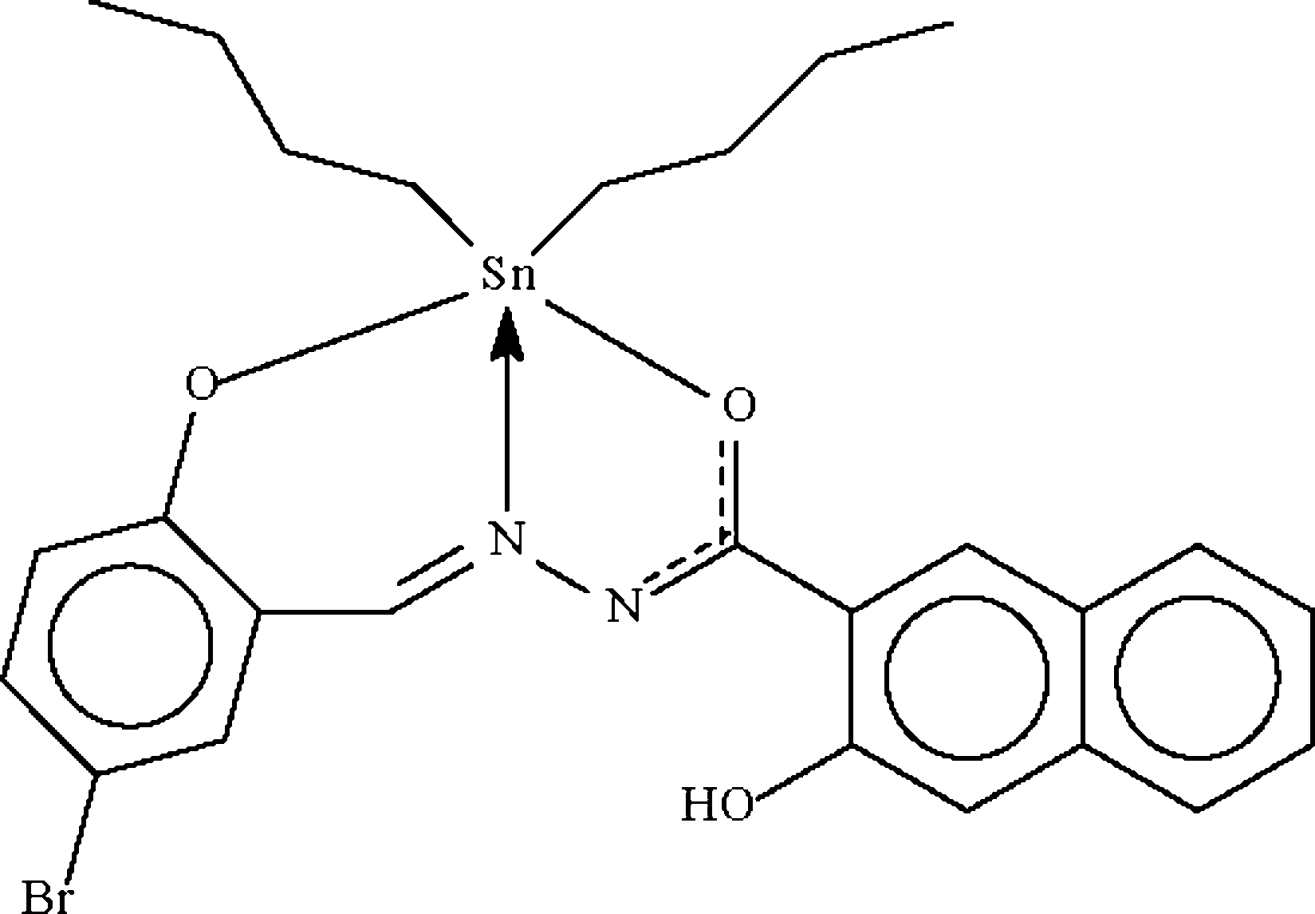


## Experimental

### 

#### Crystal data

[Sn(C_4_H_9_)_2_(C_18_H_11_BrN_2_O_3_)]
*M* *_r_* = 616.11
Triclinic, 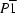

*a* = 10.1626 (2) Å
*b* = 12.2534 (2) Å
*c* = 12.5583 (2) Å
α = 62.309 (1)°
β = 83.809 (1)°
γ = 65.802 (1)°
*V* = 1256.44 (4) Å^3^
*Z* = 2
Mo *K*α radiation
μ = 2.64 mm^−1^
*T* = 140 K
0.29 × 0.26 × 0.20 mm

#### Data collection

Bruker SMART APEX diffractometer
Absorption correction: multi-scan (*SADABS*; Sheldrick, 1996 ▶) *T*_min_ = 0.515, *T*_max_ = 0.621
12053 measured reflections
5740 independent reflections
4886 reflections with *I* > 2σ(*I*)
*R*_int_ = 0.020

#### Refinement

*R*[*F* ^2^ > 2σ(*F* ^2^)] = 0.025
*wR*(*F* ^2^) = 0.060
*S* = 1.01
5740 reflections
342 parameters
47 restraints
H atoms treated by a mixture of independent and constrained refinement
Δρ_max_ = 0.53 e Å^−3^
Δρ_min_ = −0.59 e Å^−3^

### 

Data collection: *APEX2* (Bruker, 2007 ▶); cell refinement: *SAINT* (Bruker, 2007 ▶); data reduction: *SAINT*; program(s) used to solve structure: *SHELXS97* (Sheldrick, 2008 ▶); program(s) used to refine structure: *SHELXL97* (Sheldrick, 2008 ▶); molecular graphics: *X-SEED* (Barbour, 2001 ▶); software used to prepare material for publication: *publCIF* (Westrip, 2009 ▶).

## Supplementary Material

Crystal structure: contains datablocks global, I. DOI: 10.1107/S1600536809024477/xu2544sup1.cif

Structure factors: contains datablocks I. DOI: 10.1107/S1600536809024477/xu2544Isup2.hkl

Additional supplementary materials:  crystallographic information; 3D view; checkCIF report

## Figures and Tables

**Fig. 1.**
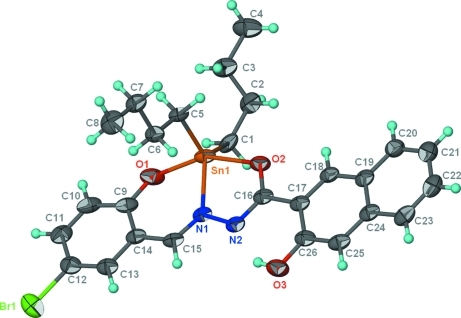
Thermal ellipsoid plot (Barbour, 2001) of Sn(C4H9)2(C18H11BrN2O3) at the 70% probability level. Hydrogen atoms are drawn as spheres of arbitrary radius. The disorder in one of the two butyl chains is not shown.

## supplementary crystallographic information

### Experimental

The Schiff base (0.39 g, 1 mmol) prepared from the condensation of 5-bromosalicylaldehyde and 3-hydroxy-2-naphthoic hydrazide was heated with dibutyltin oxide (0.25 g, 1 mmol) in ethanol (100 ml) until the oxide dissolved completely. Slow cooling of the filtrate gave the product as yellow crystals.

### Refinement

One of the two butyl chains is disordered over two positions in all four carbon atoms. The C–C distances were restrained to 1.54±0.01 Å; the anisotropic temperature factors of the carbon atoms were restrained to be nearly isotropic. The disorder refined to a 60 (1):40 (1) ratio.

Hydrogen atoms were placed at calculated positions (C–H 0.95–0.98 Å) and were treated as riding on their parent atoms, with U_iso_(H) set to 1.2–1.5U_eq_(C). The hydroxy H-atom was refined with a distance restraint of 0.84±0.01 Å.

### Crystal data

**Table:**

[Sn(C_4_H_9_)_2_(C_18_H_11_BrN_2_O_3_)]	*Z* = 2
*M**_r_* = 616.11	*F*(000) = 616
Triclinic, *P*1	*D*_x_ = 1.629 Mg m^−^^3^
Hall symbol: -P 1	Mo *K*α radiation, λ = 0.71073 Å
*a* = 10.1626 (2) Å	Cell parameters from 5075 reflections
*b* = 12.2534 (2) Å	θ = 2.5–29.6°
*c* = 12.5583 (2) Å	µ = 2.64 mm^−^^1^
α = 62.309 (1)°	*T* = 140 K
β = 83.809 (1)°	Block, yellow
γ = 65.802 (1)°	0.29 × 0.26 × 0.20 mm
*V* = 1256.44 (4) Å^3^	

### Data collection

**Table:**

Bruker SMART APEX diffractometer	5740 independent reflections
Radiation source: fine-focus sealed tube	4886 reflections with *I* > 2σ(*I*)
graphite	*R*_int_ = 0.020
ω scans	θ_max_ = 27.5°, θ_min_ = 1.8°
Absorption correction: multi-scan (*SADABS*; Sheldrick, 1996)	*h* = −13→13
*T*_min_ = 0.515, *T*_max_ = 0.621	*k* = −15→15
12053 measured reflections	*l* = −16→16

### Refinement

**Table:**

Refinement on *F*^2^	Primary atom site location: structure-invariant direct methods
Least-squares matrix: full	Secondary atom site location: difference Fourier map
*R*[*F*^2^ > 2σ(*F*^2^)] = 0.025	Hydrogen site location: inferred from neighbouring sites
*wR*(*F*^2^) = 0.060	H atoms treated by a mixture of independent and constrained refinement
*S* = 1.01	*w* = 1/[σ^2^(*F*_o_^2^) + (0.0287*P*)^2^ + 0.3063*P*] where *P* = (*F*_o_^2^ + 2*F*_c_^2^)/3
5740 reflections	(Δ/σ)_max_ = 0.001
342 parameters	Δρ_max_ = 0.53 e Å^−^^3^
47 restraints	Δρ_min_ = −0.59 e Å^−^^3^

### Fractional atomic coordinates and isotropic or equivalent isotropic displacement parameters (Å^2^)

**Table:**

	*x*	*y*	*z*	*U*_iso_*/*U*_eq_	Occ. (<1)
Sn1	0.828380 (16)	0.685444 (15)	0.242560 (14)	0.02643 (5)	
Br1	0.66105 (3)	1.14077 (3)	0.54233 (3)	0.04563 (8)	
N1	0.62849 (19)	0.86055 (18)	0.22108 (16)	0.0253 (4)	
N2	0.51208 (19)	0.88674 (18)	0.15093 (16)	0.0269 (4)	
O1	0.91148 (17)	0.75767 (16)	0.32829 (17)	0.0373 (4)	
O2	0.67073 (17)	0.70268 (15)	0.12755 (14)	0.0312 (4)	
O3	0.24653 (18)	1.01189 (17)	0.04941 (17)	0.0377 (4)	
H3	0.316 (2)	0.997 (3)	0.091 (2)	0.043 (8)*	
C1	0.9977 (13)	0.6790 (7)	0.1322 (11)	0.033 (2)	0.604 (8)
H1A	0.9610	0.7589	0.0518	0.040*	0.604 (8)
H1B	1.0748	0.6859	0.1674	0.040*	0.604 (8)
C2	1.0651 (6)	0.5515 (4)	0.1148 (4)	0.0393 (14)	0.604 (8)
H2A	0.9899	0.5474	0.0747	0.047*	0.604 (8)
H2B	1.1428	0.5585	0.0605	0.047*	0.604 (8)
C3	1.1279 (6)	0.4224 (5)	0.2312 (5)	0.0407 (14)	0.604 (8)
H3A	1.0489	0.4069	0.2811	0.049*	0.604 (8)
H3B	1.1940	0.4299	0.2774	0.049*	0.604 (8)
C4	1.2132 (13)	0.3006 (7)	0.2037 (10)	0.053 (3)	0.604 (8)
H4A	1.2446	0.2161	0.2796	0.079*	0.604 (8)
H4B	1.2983	0.3108	0.1628	0.079*	0.604 (8)
H4C	1.1501	0.2987	0.1513	0.079*	0.604 (8)
C1'	0.9820 (16)	0.6826 (9)	0.1057 (16)	0.027 (3)	0.396 (8)
H1C	0.9347	0.6928	0.0347	0.032*	0.396 (8)
H1D	1.0088	0.7599	0.0786	0.032*	0.396 (8)
C2'	1.1199 (7)	0.5504 (6)	0.1557 (8)	0.037 (2)	0.396 (8)
H2C	1.1921	0.5585	0.0961	0.044*	0.396 (8)
H2D	1.1619	0.5366	0.2307	0.044*	0.396 (8)
C3'	1.0915 (9)	0.4283 (7)	0.1826 (10)	0.047 (2)	0.396 (8)
H3C	1.0529	0.4391	0.1076	0.056*	0.396 (8)
H3D	1.0187	0.4194	0.2416	0.056*	0.396 (8)
C4'	1.2367 (15)	0.2992 (9)	0.2356 (14)	0.045 (3)	0.396 (8)
H4D	1.2299	0.2276	0.2245	0.068*	0.396 (8)
H4E	1.2546	0.2687	0.3221	0.068*	0.396 (8)
H4F	1.3167	0.3204	0.1936	0.068*	0.396 (8)
C5	0.8159 (2)	0.5186 (2)	0.4004 (2)	0.0270 (5)	
H5A	0.7944	0.4611	0.3760	0.032*	
H5B	0.9116	0.4638	0.4490	0.032*	
C6	0.7010 (2)	0.5593 (2)	0.4793 (2)	0.0284 (5)	
H6A	0.7220	0.6169	0.5039	0.034*	
H6B	0.6049	0.6132	0.4315	0.034*	
C7	0.6958 (3)	0.4372 (2)	0.5921 (2)	0.0334 (5)	
H7A	0.6758	0.3792	0.5673	0.040*	
H7B	0.7918	0.3837	0.6400	0.040*	
C8	0.5807 (3)	0.4764 (3)	0.6711 (2)	0.0438 (6)	
H8A	0.5856	0.3950	0.7443	0.066*	
H8B	0.4845	0.5231	0.6263	0.066*	
H8C	0.5978	0.5365	0.6935	0.066*	
C9	0.8505 (2)	0.8415 (2)	0.3749 (2)	0.0300 (5)	
C10	0.9369 (3)	0.8446 (2)	0.4528 (2)	0.0366 (6)	
H10	1.0369	0.7856	0.4712	0.044*	
C11	0.8800 (3)	0.9309 (2)	0.5027 (2)	0.0352 (6)	
H11	0.9402	0.9297	0.5565	0.042*	
C12	0.7347 (3)	1.0201 (2)	0.4748 (2)	0.0323 (5)	
C13	0.6465 (2)	1.0216 (2)	0.3985 (2)	0.0298 (5)	
H13	0.5475	1.0835	0.3794	0.036*	
C14	0.7019 (2)	0.9314 (2)	0.3481 (2)	0.0268 (5)	
C15	0.6026 (2)	0.9392 (2)	0.2698 (2)	0.0265 (5)	
H15	0.5080	1.0093	0.2516	0.032*	
C16	0.5445 (2)	0.8009 (2)	0.10719 (19)	0.0265 (5)	
C17	0.4293 (2)	0.8171 (2)	0.03210 (19)	0.0267 (5)	
C18	0.4605 (2)	0.7287 (2)	−0.01503 (19)	0.0265 (5)	
H18	0.5566	0.6618	−0.0006	0.032*	
C19	0.3555 (3)	0.7338 (2)	−0.08373 (19)	0.0290 (5)	
C20	0.3883 (3)	0.6414 (2)	−0.1309 (2)	0.0339 (5)	
H20	0.4847	0.5757	−0.1188	0.041*	
C21	0.2825 (3)	0.6461 (3)	−0.1935 (2)	0.0408 (6)	
H21	0.3047	0.5829	−0.2234	0.049*	
C22	0.1397 (3)	0.7457 (3)	−0.2134 (2)	0.0397 (6)	
H22	0.0665	0.7484	−0.2568	0.048*	
C23	0.1049 (3)	0.8378 (3)	−0.1720 (2)	0.0348 (5)	
H23	0.0086	0.9047	−0.1879	0.042*	
C24	0.2118 (2)	0.8345 (2)	−0.1049 (2)	0.0292 (5)	
C25	0.1806 (2)	0.9263 (2)	−0.0580 (2)	0.0304 (5)	
H25	0.0851	0.9945	−0.0731	0.036*	
C26	0.2844 (2)	0.9195 (2)	0.0086 (2)	0.0290 (5)	

### Atomic displacement parameters (Å^2^)

**Table:**

	*U*^11^	*U*^22^	*U*^33^	*U*^12^	*U*^13^	*U*^23^
Sn1	0.01985 (8)	0.01819 (8)	0.03093 (9)	−0.00410 (6)	0.00688 (6)	−0.00751 (6)
Br1	0.04928 (17)	0.05103 (18)	0.05338 (18)	−0.02710 (14)	0.01566 (14)	−0.03359 (15)
N1	0.0219 (9)	0.0205 (9)	0.0239 (9)	−0.0055 (7)	0.0040 (7)	−0.0059 (7)
N2	0.0236 (9)	0.0225 (9)	0.0251 (9)	−0.0051 (8)	0.0031 (8)	−0.0076 (8)
O1	0.0220 (8)	0.0282 (9)	0.0573 (11)	−0.0044 (7)	0.0013 (8)	−0.0209 (8)
O2	0.0257 (8)	0.0239 (8)	0.0356 (9)	−0.0017 (7)	0.0028 (7)	−0.0144 (7)
O3	0.0257 (9)	0.0317 (9)	0.0517 (11)	−0.0017 (7)	−0.0001 (8)	−0.0241 (9)
C1	0.026 (3)	0.035 (3)	0.017 (5)	−0.003 (2)	0.003 (2)	−0.005 (2)
C2	0.035 (3)	0.042 (3)	0.028 (2)	−0.008 (2)	0.009 (2)	−0.015 (2)
C3	0.033 (3)	0.034 (3)	0.043 (3)	−0.009 (2)	0.007 (2)	−0.013 (2)
C4	0.044 (4)	0.036 (3)	0.062 (6)	−0.007 (3)	0.017 (3)	−0.020 (3)
C1'	0.026 (5)	0.021 (4)	0.019 (7)	−0.007 (3)	0.010 (4)	−0.002 (3)
C2'	0.022 (3)	0.036 (4)	0.041 (4)	−0.008 (3)	0.008 (3)	−0.014 (3)
C3'	0.044 (5)	0.036 (4)	0.057 (6)	−0.016 (3)	0.016 (4)	−0.022 (4)
C4'	0.043 (6)	0.031 (4)	0.050 (6)	−0.011 (4)	0.023 (5)	−0.016 (4)
C5	0.0246 (11)	0.0182 (10)	0.0282 (11)	−0.0058 (9)	0.0018 (9)	−0.0055 (9)
C6	0.0291 (12)	0.0237 (11)	0.0279 (12)	−0.0096 (9)	0.0047 (9)	−0.0098 (9)
C7	0.0362 (13)	0.0282 (12)	0.0276 (12)	−0.0137 (11)	0.0043 (10)	−0.0065 (10)
C8	0.0462 (16)	0.0477 (16)	0.0332 (14)	−0.0236 (13)	0.0140 (12)	−0.0138 (12)
C9	0.0262 (11)	0.0193 (11)	0.0358 (13)	−0.0098 (9)	0.0044 (10)	−0.0058 (9)
C10	0.0287 (12)	0.0214 (12)	0.0467 (15)	−0.0096 (10)	−0.0042 (11)	−0.0048 (11)
C11	0.0391 (14)	0.0257 (12)	0.0362 (13)	−0.0196 (11)	−0.0023 (11)	−0.0040 (10)
C12	0.0376 (13)	0.0255 (12)	0.0331 (13)	−0.0176 (10)	0.0083 (10)	−0.0101 (10)
C13	0.0289 (12)	0.0236 (11)	0.0304 (12)	−0.0112 (10)	0.0088 (10)	−0.0085 (10)
C14	0.0257 (11)	0.0199 (11)	0.0253 (11)	−0.0087 (9)	0.0052 (9)	−0.0041 (9)
C15	0.0208 (10)	0.0189 (11)	0.0286 (11)	−0.0047 (9)	0.0054 (9)	−0.0058 (9)
C16	0.0252 (11)	0.0211 (11)	0.0235 (11)	−0.0081 (9)	0.0075 (9)	−0.0051 (9)
C17	0.0272 (11)	0.0206 (11)	0.0224 (11)	−0.0081 (9)	0.0070 (9)	−0.0048 (9)
C18	0.0249 (11)	0.0217 (11)	0.0214 (11)	−0.0056 (9)	0.0054 (9)	−0.0050 (9)
C19	0.0331 (12)	0.0248 (11)	0.0183 (10)	−0.0100 (10)	0.0064 (9)	−0.0039 (9)
C20	0.0360 (13)	0.0290 (13)	0.0267 (12)	−0.0074 (10)	0.0035 (10)	−0.0103 (10)
C21	0.0510 (16)	0.0361 (14)	0.0293 (13)	−0.0142 (12)	0.0016 (12)	−0.0130 (11)
C22	0.0425 (15)	0.0428 (15)	0.0271 (13)	−0.0187 (12)	0.0000 (11)	−0.0092 (11)
C23	0.0318 (13)	0.0340 (13)	0.0252 (12)	−0.0129 (11)	0.0025 (10)	−0.0038 (10)
C24	0.0297 (12)	0.0242 (11)	0.0217 (11)	−0.0108 (10)	0.0058 (9)	−0.0022 (9)
C25	0.0241 (11)	0.0237 (11)	0.0302 (12)	−0.0069 (9)	0.0066 (9)	−0.0058 (9)
C26	0.0290 (12)	0.0220 (11)	0.0281 (12)	−0.0087 (9)	0.0094 (10)	−0.0084 (9)

### Geometric parameters (Å, °)

**Table:**

Sn1—O1	2.0857 (17)	C5—H5B	0.9900
Sn1—C1	2.089 (15)	C6—C7	1.525 (3)
Sn1—C5	2.126 (2)	C6—H6A	0.9900
Sn1—O2	2.1531 (16)	C6—H6B	0.9900
Sn1—C1'	2.20 (2)	C7—C8	1.522 (3)
Sn1—N1	2.1932 (18)	C7—H7A	0.9900
Br1—C12	1.895 (2)	C7—H7B	0.9900
N1—C15	1.296 (3)	C8—H8A	0.9800
N1—N2	1.389 (2)	C8—H8B	0.9800
N2—C16	1.316 (3)	C8—H8C	0.9800
O1—C9	1.319 (3)	C9—C10	1.405 (3)
O2—C16	1.295 (3)	C9—C14	1.417 (3)
O3—C26	1.353 (3)	C10—C11	1.373 (4)
O3—H3	0.830 (10)	C10—H10	0.9500
C1—C2	1.538 (7)	C11—C12	1.389 (3)
C1—H1A	0.9900	C11—H11	0.9500
C1—H1B	0.9900	C12—C13	1.369 (3)
C2—C3	1.507 (6)	C13—C14	1.415 (3)
C2—H2A	0.9900	C13—H13	0.9500
C2—H2B	0.9900	C14—C15	1.429 (3)
C3—C4	1.566 (7)	C15—H15	0.9500
C3—H3A	0.9900	C16—C17	1.474 (3)
C3—H3B	0.9900	C17—C18	1.377 (3)
C4—H4A	0.9800	C17—C26	1.437 (3)
C4—H4B	0.9800	C18—C19	1.408 (3)
C4—H4C	0.9800	C18—H18	0.9500
C1'—C2'	1.538 (9)	C19—C20	1.419 (3)
C1'—H1C	0.9900	C19—C24	1.424 (3)
C1'—H1D	0.9900	C20—C21	1.367 (3)
C2'—C3'	1.516 (8)	C20—H20	0.9500
C2'—H2C	0.9900	C21—C22	1.414 (4)
C2'—H2D	0.9900	C21—H21	0.9500
C3'—C4'	1.563 (9)	C22—C23	1.359 (4)
C3'—H3C	0.9900	C22—H22	0.9500
C3'—H3D	0.9900	C23—C24	1.422 (3)
C4'—H4D	0.9800	C23—H23	0.9500
C4'—H4E	0.9800	C24—C25	1.412 (3)
C4'—H4F	0.9800	C25—C26	1.369 (3)
C5—C6	1.525 (3)	C25—H25	0.9500
C5—H5A	0.9900		
			
O1—Sn1—C1	90.6 (3)	C7—C6—H6A	109.2
O1—Sn1—C5	97.48 (8)	C5—C6—H6A	109.2
C1—Sn1—C5	126.0 (2)	C7—C6—H6B	109.2
O1—Sn1—O2	153.97 (6)	C5—C6—H6B	109.2
C1—Sn1—O2	98.7 (3)	H6A—C6—H6B	107.9
C5—Sn1—O2	96.49 (8)	C8—C7—C6	112.7 (2)
O1—Sn1—C1'	98.9 (4)	C8—C7—H7A	109.0
C1—Sn1—C1'	9.2 (6)	C6—C7—H7A	109.0
C5—Sn1—C1'	128.1 (2)	C8—C7—H7B	109.0
O2—Sn1—C1'	89.5 (4)	C6—C7—H7B	109.0
O1—Sn1—N1	82.45 (6)	H7A—C7—H7B	107.8
C1—Sn1—N1	128.2 (2)	C7—C8—H8A	109.5
C5—Sn1—N1	105.82 (7)	C7—C8—H8B	109.5
O2—Sn1—N1	72.64 (6)	H8A—C8—H8B	109.5
C1'—Sn1—N1	124.9 (2)	C7—C8—H8C	109.5
C15—N1—N2	115.59 (18)	H8A—C8—H8C	109.5
C15—N1—Sn1	128.43 (15)	H8B—C8—H8C	109.5
N2—N1—Sn1	115.90 (13)	O1—C9—C10	118.8 (2)
C16—N2—N1	112.44 (17)	O1—C9—C14	123.1 (2)
C9—O1—Sn1	133.15 (15)	C10—C9—C14	118.1 (2)
C16—O2—Sn1	115.23 (14)	C11—C10—C9	121.5 (2)
C26—O3—H3	110 (2)	C11—C10—H10	119.3
C2—C1—Sn1	114.9 (8)	C9—C10—H10	119.3
C2—C1—H1A	108.5	C10—C11—C12	120.1 (2)
Sn1—C1—H1A	108.5	C10—C11—H11	119.9
C2—C1—H1B	108.5	C12—C11—H11	119.9
Sn1—C1—H1B	108.5	C13—C12—C11	120.5 (2)
H1A—C1—H1B	107.5	C13—C12—Br1	120.52 (18)
C3—C2—C1	113.6 (6)	C11—C12—Br1	118.98 (18)
C3—C2—H2A	108.8	C12—C13—C14	120.4 (2)
C1—C2—H2A	108.8	C12—C13—H13	119.8
C3—C2—H2B	108.8	C14—C13—H13	119.8
C1—C2—H2B	108.8	C13—C14—C9	119.4 (2)
H2A—C2—H2B	107.7	C13—C14—C15	116.9 (2)
C2—C3—C4	110.1 (6)	C9—C14—C15	123.7 (2)
C2—C3—H3A	109.6	N1—C15—C14	126.7 (2)
C4—C3—H3A	109.6	N1—C15—H15	116.7
C2—C3—H3B	109.6	C14—C15—H15	116.7
C4—C3—H3B	109.6	O2—C16—N2	123.8 (2)
H3A—C3—H3B	108.2	O2—C16—C17	118.5 (2)
C2'—C1'—Sn1	111.5 (10)	N2—C16—C17	117.68 (19)
C2'—C1'—H1C	109.3	C18—C17—C26	118.6 (2)
Sn1—C1'—H1C	109.3	C18—C17—C16	118.82 (19)
C2'—C1'—H1D	109.3	C26—C17—C16	122.6 (2)
Sn1—C1'—H1D	109.3	C17—C18—C19	122.5 (2)
H1C—C1'—H1D	108.0	C17—C18—H18	118.7
C3'—C2'—C1'	112.8 (8)	C19—C18—H18	118.7
C3'—C2'—H2C	109.0	C18—C19—C20	122.1 (2)
C1'—C2'—H2C	109.0	C18—C19—C24	118.3 (2)
C3'—C2'—H2D	109.0	C20—C19—C24	119.6 (2)
C1'—C2'—H2D	109.0	C21—C20—C19	120.5 (2)
H2C—C2'—H2D	107.8	C21—C20—H20	119.7
C2'—C3'—C4'	109.0 (7)	C19—C20—H20	119.7
C2'—C3'—H3C	109.9	C20—C21—C22	119.6 (2)
C4'—C3'—H3C	109.9	C20—C21—H21	120.2
C2'—C3'—H3D	109.9	C22—C21—H21	120.2
C4'—C3'—H3D	109.9	C23—C22—C21	121.5 (2)
H3C—C3'—H3D	108.3	C23—C22—H22	119.3
C3'—C4'—H4D	109.5	C21—C22—H22	119.3
C3'—C4'—H4E	109.5	C22—C23—C24	120.4 (2)
H4D—C4'—H4E	109.5	C22—C23—H23	119.8
C3'—C4'—H4F	109.5	C24—C23—H23	119.8
H4D—C4'—H4F	109.5	C25—C24—C23	122.6 (2)
H4E—C4'—H4F	109.5	C25—C24—C19	119.1 (2)
C6—C5—Sn1	113.65 (14)	C23—C24—C19	118.3 (2)
C6—C5—H5A	108.8	C26—C25—C24	121.6 (2)
Sn1—C5—H5A	108.8	C26—C25—H25	119.2
C6—C5—H5B	108.8	C24—C25—H25	119.2
Sn1—C5—H5B	108.8	O3—C26—C25	118.2 (2)
H5A—C5—H5B	107.7	O3—C26—C17	121.9 (2)
C7—C6—C5	112.22 (19)	C25—C26—C17	119.9 (2)
			
O1—Sn1—N1—C15	−9.94 (19)	C9—C10—C11—C12	−1.4 (4)
C1—Sn1—N1—C15	−94.8 (4)	C10—C11—C12—C13	0.8 (4)
C5—Sn1—N1—C15	85.7 (2)	C10—C11—C12—Br1	−178.50 (18)
O2—Sn1—N1—C15	177.7 (2)	C11—C12—C13—C14	0.6 (3)
C1'—Sn1—N1—C15	−105.6 (5)	Br1—C12—C13—C14	179.89 (16)
O1—Sn1—N1—N2	173.53 (15)	C12—C13—C14—C9	−1.4 (3)
C1—Sn1—N1—N2	88.7 (4)	C12—C13—C14—C15	179.8 (2)
C5—Sn1—N1—N2	−90.79 (15)	O1—C9—C14—C13	−177.6 (2)
O2—Sn1—N1—N2	1.18 (13)	C10—C9—C14—C13	0.8 (3)
C1'—Sn1—N1—N2	77.9 (5)	O1—C9—C14—C15	1.1 (4)
C15—N1—N2—C16	−178.12 (19)	C10—C9—C14—C15	179.5 (2)
Sn1—N1—N2—C16	−1.1 (2)	N2—N1—C15—C14	178.17 (19)
C1—Sn1—O1—C9	146.5 (3)	Sn1—N1—C15—C14	1.6 (3)
C5—Sn1—O1—C9	−87.0 (2)	C13—C14—C15—N1	−175.1 (2)
O2—Sn1—O1—C9	34.9 (3)	C9—C14—C15—N1	6.1 (4)
C1'—Sn1—O1—C9	142.4 (3)	Sn1—O2—C16—N2	0.9 (3)
N1—Sn1—O1—C9	18.1 (2)	Sn1—O2—C16—C17	−178.28 (14)
O1—Sn1—O2—C16	−18.6 (2)	N1—N2—C16—O2	0.2 (3)
C1—Sn1—O2—C16	−128.4 (3)	N1—N2—C16—C17	179.36 (17)
C5—Sn1—O2—C16	103.52 (16)	O2—C16—C17—C18	−0.9 (3)
C1'—Sn1—O2—C16	−128.1 (3)	N2—C16—C17—C18	179.84 (19)
N1—Sn1—O2—C16	−1.07 (14)	O2—C16—C17—C26	177.4 (2)
O1—Sn1—C1—C2	137.0 (6)	N2—C16—C17—C26	−1.8 (3)
C5—Sn1—C1—C2	37.3 (8)	C26—C17—C18—C19	−0.9 (3)
O2—Sn1—C1—C2	−67.4 (7)	C16—C17—C18—C19	177.48 (19)
C1'—Sn1—C1—C2	−69 (3)	C17—C18—C19—C20	−179.3 (2)
N1—Sn1—C1—C2	−142.1 (5)	C17—C18—C19—C24	0.1 (3)
Sn1—C1—C2—C3	−59.5 (10)	C18—C19—C20—C21	177.6 (2)
C1—C2—C3—C4	−171.9 (8)	C24—C19—C20—C21	−1.8 (3)
O1—Sn1—C1'—C2'	85.7 (8)	C19—C20—C21—C22	1.3 (4)
C1—Sn1—C1'—C2'	59 (2)	C20—C21—C22—C23	0.2 (4)
C5—Sn1—C1'—C2'	−21.2 (11)	C21—C22—C23—C24	−1.2 (4)
O2—Sn1—C1'—C2'	−119.1 (8)	C22—C23—C24—C25	−178.8 (2)
N1—Sn1—C1'—C2'	172.7 (6)	C22—C23—C24—C19	0.6 (3)
Sn1—C1'—C2'—C3'	67.2 (12)	C18—C19—C24—C25	0.9 (3)
C1'—C2'—C3'—C4'	−178.6 (11)	C20—C19—C24—C25	−179.7 (2)
O1—Sn1—C5—C6	68.73 (16)	C18—C19—C24—C23	−178.6 (2)
C1—Sn1—C5—C6	165.0 (4)	C20—C19—C24—C23	0.8 (3)
O2—Sn1—C5—C6	−89.24 (16)	C23—C24—C25—C26	178.5 (2)
C1'—Sn1—C5—C6	176.3 (5)	C19—C24—C25—C26	−1.0 (3)
N1—Sn1—C5—C6	−15.50 (18)	C24—C25—C26—O3	179.3 (2)
Sn1—C5—C6—C7	−179.63 (15)	C24—C25—C26—C17	0.1 (3)
C5—C6—C7—C8	−179.5 (2)	C18—C17—C26—O3	−178.3 (2)
Sn1—O1—C9—C10	164.47 (17)	C16—C17—C26—O3	3.3 (3)
Sn1—O1—C9—C14	−17.1 (3)	C18—C17—C26—C25	0.8 (3)
O1—C9—C10—C11	179.1 (2)	C16—C17—C26—C25	−177.5 (2)
C14—C9—C10—C11	0.6 (4)		

### Hydrogen-bond geometry (Å, °)

**Table:**

*D*—H···*A*	*D*—H	H···*A*	*D*···*A*	*D*—H···*A*
O3—H3···N2	0.83 (1)	1.88 (2)	2.606 (2)	146 (3)
